# Frontotemporal dementia as a comorbidity to idiopathic normal pressure hydrocephalus (iNPH): a short review of literature and an unusual case

**DOI:** 10.1186/s12987-017-0060-7

**Published:** 2017-04-19

**Authors:** V. E. Korhonen, E. Solje, N. M. Suhonen, T. Rauramaa, R. Vanninen, A. M. Remes, V. Leinonen

**Affiliations:** 1 Department of Neurosurgery, Kuopio University Hospital, P.O. Box 100, 70029 KYS Kuopio, Finland; 2 University of Eastern Finland, P.O. Box 100, 70029 KYS Kuopio, Finland; 3 Institute of Clinical Medicine-Neurology, University of Eastern Finland, P.O. Box 1627, 70211 Kuopio, Finland; 4 Medical Research Center, Oulu University Hospital, P.O. Box 20, 90029 Oulu, Finland; 5 Unit of Clinical Neuroscience, Neurology, University of Oulu, P.O. Box 5000, 90014 Oulu, Finland; 6 Institute of Clinical Medicine-Pathology, School of Medicine, University of Eastern, Kuopio, Finland; 7 Department of Pathology, Kuopio University Hospital, P.O. Box 162, 70211 Kuopio, Finland; 8 Department of Radiology, Kuopio University Hospital, P.O. Box 100, 70029 KYS Kuopio, Finland

**Keywords:** *C9ORF72* repeat expansion, CSF shunt, Dementia, Idiopathic normal pressure hydrocephalus, Neurodegeneration

## Abstract

Behavioural variant frontotemporal dementia (bvFTD) and idiopathic normal pressure hydrocephalus (iNPH) are neurodegenerative diseases that can present with similar symptoms. These include decline in executive functions, psychomotor slowness, and behavioural and personality changes. Ventricular enlargement is a key radiological finding in iNPH that may also be present in bvFTD caused by the *C9ORF72* expansion mutation. Due to this, bvFTD has been hypothesized as a potential comorbidity to iNPH but bvFTD patients have never been identified in studies focusing in clinical comorbidities with iNPH. Here we describe a patient with the *C9ORF72* expansion-associated bvFTD who also showed enlarged ventricles on brain imaging. The main clinical symptoms were severe gait disturbances and psychiatric problems with mild cognitive decline. Cerebrospinal fluid removal increased the patient’s walking speed, so a ventriculoperitoneal shunt was placed. After insertion of the shunt, there was a significant improvement in walking speed as well as mild improvement in cognitive function but not in neuropsychiatric symptoms relating to bvFTD. Comorbid iNPH should be considered in bvFTD patients who have enlarged ventricles and severely impaired gait.

## Background

Idiopathic normal pressure hydrocephalus (iNPH) is a progressive neurodegenerative disease that has three core symptoms: gait disturbance, cognitive impairment, and urinary incontinence [1]. Patients with iNPH may show partial or full expression of this triad of symptoms as well as impaired frontal executive function [2]. Ventriculomegaly is observed along with the clinical symptoms [3, 4].

The most common comorbidities among iNPH patients are hypertension, Alzheimer’s disease (AD) and vascular dementia [3, 5]. Amyloid beta plaques and hyperphosphorylated tau-protein inclusions have been frequently identified in neuropathological studies and studies using frontal cortical biopsies in iNPH [6–8].

Although frontotemporal dementia (FTD) has been listed routinely as a comorbidity [3, 9] in iNPH, none of the studies focusing on the comorbidities in iNPH have identified patients suffering from both of the diseases concomitantly [3, 5, 6]. In addition, none of the studies evaluating the neuropathology of iNPH patients have included, to our knowledge, immunohistochemical staining of p62 or TDP-43, which are indicative neuropathology in about half of cases with frontotemporal lobar degeneration (FTLD) spectrum.

FTLD encompasses a clinically and genetically heterogeneous group of neurodegenerative diseases. The most frequent clinical subtype is behavioural variant frontotemporal dementia (bvFTD), which presents as progressive changes in personality and behaviour and deficits in executive functions [10]. Also gait disturbance, psychomotor slowing with extrapyramidal symptoms as well as urinary incontinence may be present.

Up to 50% of FTLD cases are familial. Mutations in microtubule-associated protein tau (*MAPT*), progranulin (*PGRN*), and expanded hexanucleotide repeat in a non-coding region of the chromosome 9 open reading frame 72 (*C9ORF72*) are the most common genetic causes of FTLD, together accounting for 60% of familial FTLD cases [11]. The neuropathology associated with FTLD is heterogeneous, which is not surprising considering the extensive variability seen in the clinical features and in genetics. Five major histopathological subtypes of FTLD are now recognized, but the two most common neuropathological findings are tau- or TDP-43 positive inclusions [12]. Mutations in *MAPT* are associated with tau-pathology and mutations in *PGRN* or the *C9ORF72* expansion are typically associated with tau-negative but TDP-43 positive neuropathology [11, 13, 14]. The *C9ORF72* expansion is the major genetic cause of FTLD [15, 16]. In Finland, the *C9ORF72* repeat expansion has been reported to be the highest in the world and it has been determined to be 48% in familial FTLD [16, 17]. The most common motor presentation associated with the *C9ORF72* expansion is amyotrophic lateral sclerosis (ALS) but extrapyramidal symptoms have also been reported [18].

Here we describe a patient with the *C9ORF72* expansion-associated bvFTD who presented with severe gait disturbances and ventriculomegaly on brain imaging. The patient’s walking speed increased 73% after the insertion of a ventriculoperitoneal shunt, and mild cognitive improvement was also detected. In addition, the patient subjectively experienced a significant relief of her symptoms in terms of her memory and her ability to move.

## Patient

A 59-year-old woman without any previous history of neurological or psychiatric diseases was referred to a neurologist due to delusions and declining performance at work. Compulsive behaviour, apathy, and hyperorality were the main neuropsychiatric symptoms. ALS and unspecified dementia had been diagnosed in first-degree relatives. Her Mini Mental Status Examination (MMSE) score was 26/30. In the neuropsychological examination, the most prominent dysfunction was found in executive function (e.g. in verbal fluency, on the clock drawing test, on the Trail Making Test B, and on the Stroop test) and in psychomotor speed (Trail Making Test A and the Digit Symbol test). In addition, her performance was poor on tests of visuospatial skills, which can be due to executive dysfunction rather than to visuospatial deficits per se [19]. The patient performed relatively well on verbal memory tests but had deterioration in her visual memory. On the frontal behavioral inventory (FBI), her next-of-kin reported mild behavioural changes, mainly apathy. Her BDI-II score did not indicate depression. BvFTD was diagnosed based on the clinical assessment, and the presence of the *C9ORF72* repeat expansion (>60 repeats) confirmed the diagnosis.

Risperidone was prescribed as a treatment for her delusions, but the patient had begun to suffer from bradykinesia and shortened step length. Psychomotor slowness was also evident. Due to the extrapyramidal symptoms, risperidone was replaced with aripiprazole. Despite the mild conservative antipsychotic treatment, the patient’s extrapyramidal symptoms continued to worsen. Three years after her first contact with a neurologist, the patient began to suffer from daily urinary incontinence. [(123)I]β-CIT SPECT revealed normal I-123 uptake, excluding idiopathic Parkinson’s disease as the cause of the extrapyramidal symptoms.

**Fig. 1:**
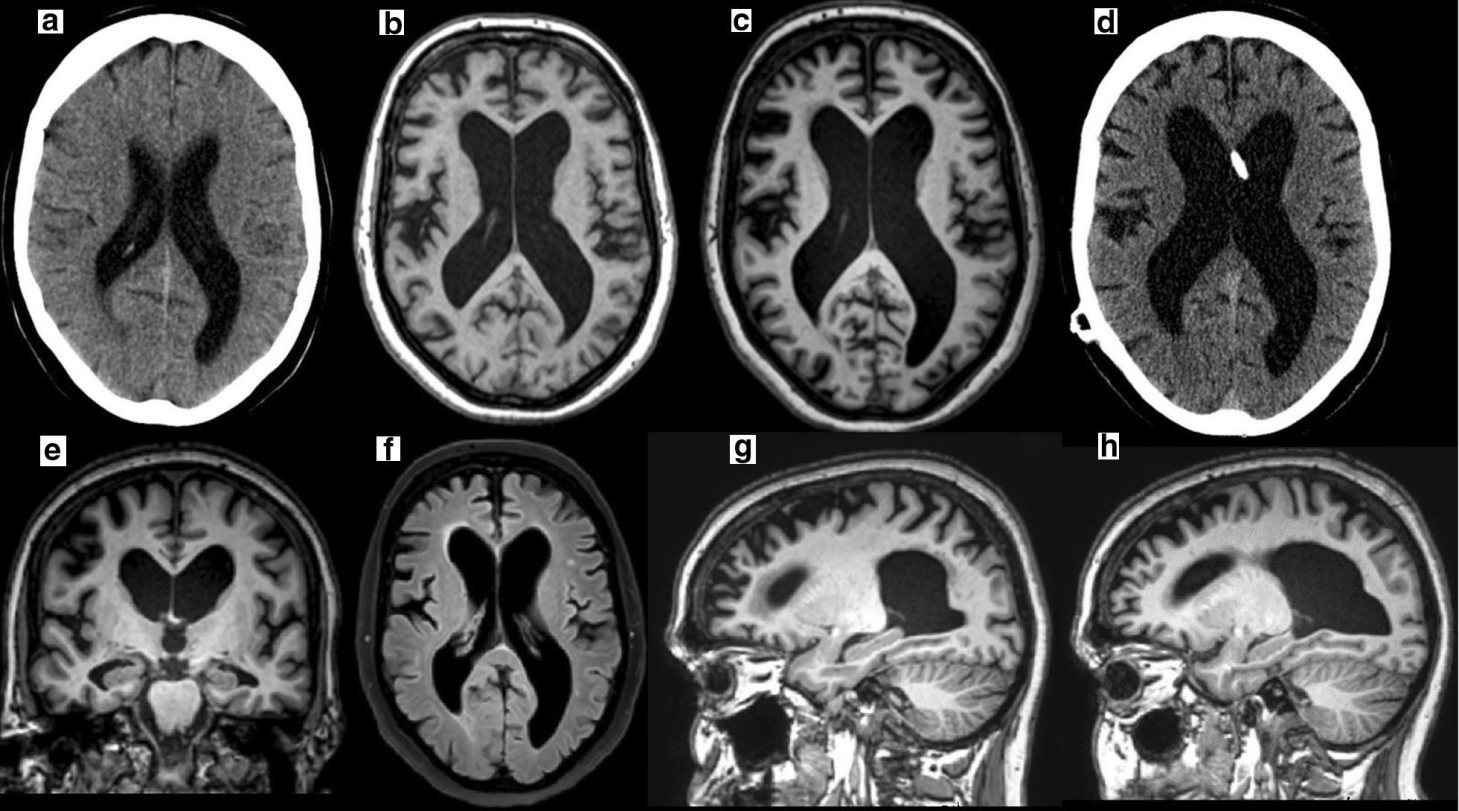
**a** A brain CT scan that was performed because of an episode of syncope 5 years before the onset of symptoms shows no atrophy (Evans’ index 0.33), but some asymmetry of the occipital horns of the lateral ventricles is apparent. **b** The first T1weighed MRI, which was performed 1 year after the onset of symptoms, shows the development of frontal cortical atrophy and central atrophy (Evans’ index 0.42) **c** with some progression over the follow-up time of 1 year (Evans’ index 0.45). **d** The CT control scan that was performed 6 months after shunt placement shows stable ventricular size (Evans’ index 0.45). **e** The coronal slice shows hippocampal atrophy (Scheltens grade 2/4) but no disproportionate obliteration of the parasagittal sulci. **f** The FLAIR sequence shows only minor changes in the white matter. The parasagittal images show that frontal cortical atrophy is more prominent on the *right* (**g**) compared to the *left side* (**h**)

MRI was performed 1 year after the onset of the first symptoms and it revealed predominantly frontal cortical atrophy and dilatation of the lateral ventricles. The parasagittal sulci were not obliterated. Hippocampal atrophy (Scheltens grade 2/4) was also present. White matter changes were mild (Fig. 1).

**Fig. 2:**
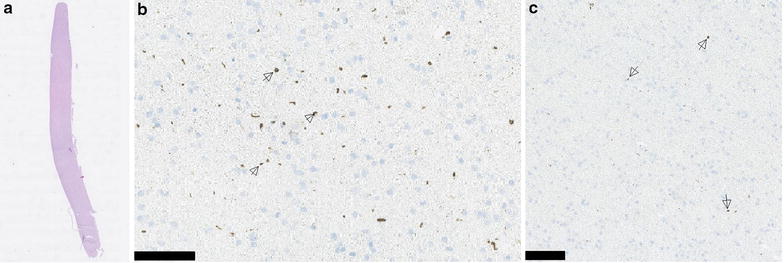
**a** Hematoxylin and eosin staining of a 1.6 cm biopsy of the frontal cortex that contained both grey and white matter. **b** Abundant TDP-43 pathology (*arrows*) was present, mostly in the form of thin neurites and varying shapes of inclusions, in both grey and white matter. *Scale bar* 100 µm. **c** There was moderate p62 (*arrows*) staining, mostly in thin neurites. *Scale bar* 100 µm

Despite her genetically confirmed bvFTD, the patient was referred to a neurosurgeon for iNPH evaluation due to gait difficulties, psychomotor slowness, urinary incontinence, and ventricular enlargement. The MMSE score had decreased from 26/30 to 23/30. The lumbar CSF removal (tap test) showed a 15% increase in walking speed, and the patient subjectively experienced significant relief from her gait-related symptoms. A ventriculoperitoneal shunt was placed 3 years after the onset of the first symptoms, and a frontal cortical biopsy was taken during the insertion of the intraventricular catheter. The biopsy included both white and grey matter. Immunohistochemical staining revealed a moderate level of p62 and TDP-43 immunoreactive lesions, mainly in the form of thin neurites (Fig. 2). Neither beta-amyloid nor hyperphosphorylated-tau was present.

The response to the shunt was evaluated 3 and 12 months after its implantation. At the 3-month evaluation, the patient’s walking speed in two 10 m walk tests had increased 73% from 0.18 to 0.30 m/s. Cognition was evaluated using the CERAD-NB. Improvement was seen in the MMSE score, which increased from 23/30 to 26/30, and in the score for the clock drawing test [20], which increased from 3/6 to 6/6. However, the patient’s word list learning continued to slowly deteriorate. There were no notable differences between pre-shunt and post-shunt findings for the other subtests of the CERAD-NB. Insertion of the ventriculoperitoneal shunt did not have effect on the patient’s urinary incontinence. At the 12-month evaluation, the patient was still able to walk without any walking aids and her walking speed had remained unchanged compared to the 3-month evaluation. The patient’s MMSE score had decreased to pre-operative level (22/30) and also the neuropsychiatric symptoms including delusions, hyperorality, increased sex drive and disinhibition were more prominent. The urinary incontinence had remained unchanged.

## Discussion and conclusions

This is the first report of a response to a shunt in a patient with genetically, pathologically, and clinically confirmed bvFTD. Due to the patient’s prominent gait difficulties, psychomotor slowness, urinary incontinence, and enlarged ventricles, iNPH was suspected.

In iNPH gait improvement is the most prominent, frequent, and usually the earliest sign of response to a shunt compared to improvements in urinary incontinence or cognition [21]. The marked improvement of walking speed and ability to move was also seen in our patient.

A slight improvement was also detected in patient’s cognitive functions after the shunt surgery. A recent meta-analysis by Peterson et al. [22] found strong evidence for improvement in iNPH patients following shunt surgery for global cognitive measure (MMSE), verbal learning and memory, and psychomotor speed. The evidence for improvement was weaker for tests of executive function. In the present case, MMSE score improved significantly. In addition, the patient showed improvement in the clock drawing subtest of the CERAD-NB, indicating possible improvement in executive functioning. However, the shunt surgery did not have a positive effect on verbal learning or memory as measured by the CERAD-NB. The placement of the ventriculoperitoneal shunt did not have any significant effect on the urinary incontinence or the behavioural symptoms associated with bvFTD.

The differential diagnosis between bvFTD and iNPH maybe challenging due to similar clinical symptoms in both conditions. Extrapyramidal symptoms, including rigidity and bradykinesia are typical in patients with bvFTD [18]. Gait and balance disturbance as well as psychomotor slowing are prominent symptoms in iNPH [1]. Deficits in executive functions are core cognitive changes in both iNPH and bvFTD [1, 10] and neuropsychiatric symptoms are frequently detected in both diseases [23, 24]. Also, psychiatric manifestations such as depression, mania, aggression, disturbances of impulse control, obsessive–compulsive disorder, and psychosis, including paranoia and hallucinations may be seen in patients with iNPH and bvFTD [3, 9, 25]. Neuroradiological findings may also be similar in both diseases. A frontal and temporal brain atrophy is the most common feature in the *C9ORF72* expansion associated bvFTD. However, sometimes central atrophy with prominent ventricular enlargement mimicking iNPH may also be present in bvFTD [18, 26].

The etiology of iNPH is still largely unknown, but the most common neuropathologies in iNPH are vascular and AD-related changes [7]. There is also increasing evidence that iNPH may have a genetic component [27–31]. We have previously observed that over 40% of iNPH patients present, on their cortical biopsies, with either beta-amyloid, hyperphosphorylated tau protein or both [8]. It has been reported that beta-amyloid and hyperphosphorylated tau protein accumulation in the brain is increased in Kaolin-aged hydrocephalic rats and it has been hypothesized that the accumulation is due to decreased CSF clearance [32, 33]. Therefore, it might be that part of the shunt response seen in iNPH patients could be due to improved clearance of solute neurotoxic molecules in the brain. However, we do not believe that already existing aggregates could be cleared from the brain. In the presented case, the shunt response is most likely due to decrease in the pressure gradient. This hypothesis is further supported by the observation that the patient’s neuropsychiatric symptoms related to bvFTD worsened over time and therefore we did not observe any effect that that the potential improved CSF clearance of TDP-43 or p62 had on the shunt response in the described patient.

The present case illustrates that comorbid iNPH should be considered in bvFTD patients who have enlarged ventricles and severely impaired gait and that the relationship between iNPH and *C9ORF72* expansion-associated bvFTD and the frequency of the comorbidity merit further evaluation.

## Abbreviations

AD: Alzheimer’s disease
ALS: amyotrophic lateral sclerosis
bvFTD: behavioral variant frontotemporal dementia
CERAD-NB: Consortium to Establish a Registry for Alzheimer’s disease Neuropsychological Battery
*CHMP2B*: chromatin-modifying protein 2B
CSF: Cerebrospinal fluid
FBI: frontal behavioral inventory
FTLD: frontotemporal lobar degeneration
*GRN*: progranulin
iNPH: idiopathic normal pressure hydrocephalus
*MAPT*: microtubule-associated protein tau
p62: sequestosome-1/ubiquitin-binding protein
TDP-43: TAR DNA binding protein 43
